# Variation in Assisted Living Regulations Within and Across States

**DOI:** 10.1093/geroni/igaa057.2523

**Published:** 2020-12-16

**Authors:** Paula Carder, Lindsey Smith, Taylor Bucy, Jaclyn Winfree, Wenhan Zhang, Kali Thomas

**Affiliations:** 1 Portland State University, Portland, Oregon, United States; 2 PSU School of Public Health, Portland, Oregon, United States; 3 Brown University, Providence, Rhode Island, United States

## Abstract

Assisted living (AL) regulations have been long recognized as being highly variable across states. A new approach developed by our team, Health Services Regulatory Analysis, allows for a more granular identification of within-state variation in AL regulation. We identified 172 licensing classifications from the 50 states and DC representing 58 primary license types, 48 sub-types, and 66 designations that can modify a primary or sub-license. Over two-thirds (72%) of dementia-specific classifications require that all staff receive initial dementia training, compared to only one-third (33%) of general AL classifications. This trend is similarly reflected in cognitive-screening requirements, present in 67% of dementia-specific classifications and 42% of general AL classifications. Regulatory theory describes how licensing agencies respond to various forces and values. Within-state AL regulatory variation reflects a combination of oversight mandates, population-specific needs (e.g., people with dementia), historic policies, and provider influence, with implications for consumers, policy-makers and researchers. Part of a symposium sponsored by Assisted Living Interest Group.

